# Measuring Stress Reduction in Patients Receiving Multimedia Entertainment During Vascular Surgery Under Regional Anesthesia: Protocol for a Randomized Controlled Study

**DOI:** 10.2196/70597

**Published:** 2025-07-11

**Authors:** Hagen Kerndl, Thomas Peter Millian, Karlheinz Gürtler, Alexander Hyhlik-Duerr

**Affiliations:** 1 Department of Vascular Surgery Faculty of Medicine University of Augsburg Augsburg Germany; 2 Department of Anesthesiology and Operative Intensive Care Medicine Faculty of Medicine University of Augsburg Augsburg Germany

**Keywords:** audiovisual distraction, arteriosclerosis, video goggles, vascular surgery, patient comfort

## Abstract

**Background:**

This study explores the treatment of internal carotid artery stenosis in patients with a high risk for cardiovascular events. The use of regional anesthesia permits ongoing neurological monitoring and enhances patient safety during the procedure. However, the operation can be stressful and lengthy, highlighting the need for strategies to alleviate patient discomfort. This study evaluates audiovisual distractions such as video goggles to potentially improve patient experiences during carotid surgeries, a topic that has not yet been comprehensively researched.

**Objective:**

We aimed to (1) determine whether there is a benefit for patients regarding stress reduction when using video goggles during vascular surgery of the carotid artery and (2) determine whether any parameters can effectively measure such a potential benefit.

**Methods:**

This prospective, randomized study at the University Hospital Augsburg is evaluating the use of HappyMed video goggles by patients undergoing carotid endarterectomy under regional anesthesia. Participants are randomized into either the intervention group, which receives the video goggles, or the control group, which does not. The surgical and anesthetic procedures remain consistent across both groups. Patients are eligible if they meet the surgical criteria, are able to lie supine, and are cooperative. Patients are excluded if they are receiving corticosteroids, have dementia, or have a language barrier. The study uses questionnaires and vital/laboratory parameters, including cortisol levels and heart rate, to assess stress and anxiety. To monitor potential motion sickness, the occurrence of nausea or vomiting is documented. Both patients and surgical staff will evaluate the experience postoperatively to determine the goggles’ impact on patient experience and stress management during surgery.

**Results:**

The study has been approved by the local ethics committee and is registered at ClinicalTrials.gov. Patient inclusion started in September 2022 and should be completed within 3 to 4 years. This paper presents a study protocol that was finalized and approved by the local ethics committee in September 2022. At the time of this protocol’s final submission for publication, approximately 90% of the recruitment had been completed. Following completion of recruitment and data acquisition, the results are intended to be published within one year.

**Conclusions:**

This study aims to improve patient comfort and perioperative care during vascular surgery. Therefore, our study aims to investigate if the use of video goggles during surgery is feasible and if there are parameters that indicate a benefit for patients. This study is being conducted as a pilot trial to provide a foundation for future research aimed at improving patient comfort during carotid artery surgery under local anesthesia.

**Trial Registration:**

ClinicalTrials.gov NCT06704230; https://clinicaltrials.gov/study/NCT06704230

**International Registered Report Identifier (IRRID):**

DERR1-10.2196/70597

## Introduction

Atherosclerosis and macroangiopathy of the internal carotid artery are common conditions in patients with elevated cardiovascular risk. Treatment of internal carotid artery stenosis is often conservative, involving risk factor modification and medication. However, when a certain degree of stenosis is reached or symptoms suggestive of stroke are present, thrombendarteriectomy and revascularization is indicated. Revascularization can be performed either through interventional angiography with stent placement or open surgical endarterectomy [1-3]. The operation is frequently carried out under regional plexus anesthesia, which allows the patient to be awake and enables continuous neuromonitoring. This approach offers the advantage of monitoring for neurological changes during carotid artery clamping, allowing the surgical team to immediately respond, for example, by placing a shunt system to ensure cerebral antegrade perfusion [4]. As a result, regional anesthesia provides a benefit during surgery. However, for patients, the procedure, which can last up to 2 hours or more in some cases, may pose a significant burden. The fixed position, inability to move, sterile drapes over the face, manipulation by the surgical team, and anxiety about potential complications are just a few of the factors that may distress patients during this operation [4]. All in all, studies of operations with local or general anesthesia show that neither technique has any advantage over the other [4]. Both types are carried out frequently. Increased sweating and reports of substantial subjective distress are not uncommon if the procedure is performed under local anesthesia. To the authors’ knowledge, there have been no previous attempts to quantify stress parameters or to reduce stress in these patients.

In many medical fields, devices and therapies are now being used to reduce patient stress in the perioperative setting. In procedures performed under local or regional anesthesia, such as in orthopedics or dentistry, efforts are being made to make operations more tolerable and less stressful for patients [5-7]. For example, music and video goggles can be used to entertain and distract patients during the intervention. Several studies have shown that music can significantly reduce patients’ anxiety, stress, and perception of pain [6,8]. These applications, however, are often limited to dental or pediatric patients [9,10]. In these patient groups, a need for anxiety reduction is understandable, as many individuals find dental treatment stressful. Nonpharmacological sedation or improving the experience for children is highly justifiable. Newer approaches using video goggles appear in more and more fields, but good systematic randomized studies have not yet been performed. In vascular surgery, particularly carotid surgery, the use of audiovisual distraction during the procedure has not been implemented, to our knowledge, and its potential benefits remain undocumented. Because of the special setting of the surgery and the burden on patients, it is necessary to analyze the use of audiovisual distraction devices in carotid surgery. This study aims to demonstrate the feasibility and potential patient benefit of using video goggles in vascular surgery, particularly during carotid procedures. Additionally, we seek to assess a range of parameters that may be suitable for capturing such effects.

## Methods

### Trial Design and Study Population

In this prospective, randomized study, the use of the HappyMed video goggles (HappyMed GmbH) will be evaluated in patients undergoing carotid endarterectomy under regional anesthesia. The study is being conducted as a single-center study at the University Hospital Augsburg (Augsburg, Germany) in collaboration with the departments of vascular surgery and anesthesiology. Patient acquisition started in September 2022 and should be completed within three years. All patients scheduled for the abovementioned procedure who meet the inclusion criteria are informed about the study and invited to participate. Upon agreeing to participate, patients are randomized according to a predefined list, assigning them to either the intervention group (with video goggles) or control group (without video goggles). The surgical procedure is identical for both groups, and the study does not influence the operative or anesthetic management of the patients.

### Ethical Considerations

Informed consent for the study is obtained during preoperative preparation, separate from the surgical consent process, to ensure that patients clearly distinguish between their participation in the study and the surgical procedure. The study was submitted to the responsible ethics committee (Ethikkommission Ludwig Maximilians Universität München, Germany) for review and has been approved (reference number 21-1199). Both the sponsor (University Hospital Augsburg) and the funder (HappyMed GmbH) have no direct influence on data management or the publication of the study results. All data are fully anonymized and treated confidentially; no data will be shared with third parties. No compensation was provided to participants.

### Inclusion Criteria

Patients are included who undergo carotid endarterectomy under regional anesthesia. For anesthesia, a cervical block is used. To qualify for the study, patients must meet the clinical requirements for the surgery, be sufficiently oriented and cooperative, and be able to lie supine for the duration of the procedure.

### Exclusion Criteria

Exclusion criteria include current corticosteroid use or medications affecting cortisol levels, as well as patients with insurmountable language barriers that would impair understanding of the questionnaire. Additionally, patients with severe dementia or lacking the capacity to provide informed consent are excluded.

### Secondary Exclusion Criteria and Adverse Events

Patients who withdraw consent after enrollment will be excluded from the study. If complications arise during surgery requiring conversion to general anesthesia, patients will also be excluded.

### Randomization

Patient randomization follows a predefined list based on patient ID, assigning them to either the control or intervention group. The list is followed sequentially, and random assignment to either the intervention or control group is performed using Microsoft Excel. The randomization scheme is constructed to ensure a 1:1 allocation ratio (50-50) upon reaching a total sample size of 100 patients. Due to the nature of the intervention, blinding of participants and investigators is not feasible.

### Sample Size Estimation

Due to the exploratory nature of the study, no formal sample size estimation was performed. There are no other studies covering this topic. Therefore, there are no data available on how great a relevant effect should be, nor on how large the sample size should be. The goal is to use this study and its preliminary results as a basis for initiating a confirmatory trial. With a sample size of 100 patients, the sample should be big enough to provide an overview on the possible characteristic values.

### Interventions

**Figure 1:**
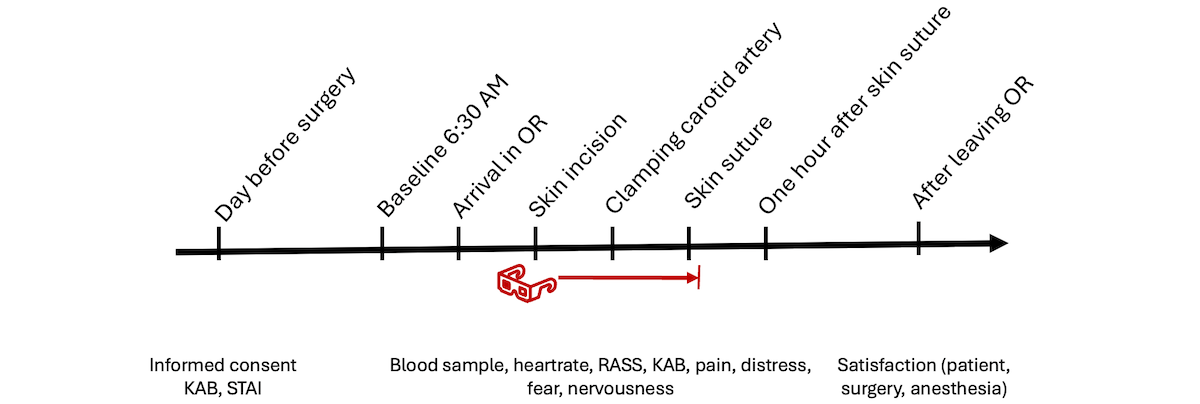
Timing and measured parameters during surgery. The intervention group uses the goggles during the time indicated by the red line. KAB: Kurzfragebogen zur aktuellen Beanspruchung (German for “short questionnaire for acute stress”); OR: operation room; RASS: Richmond Agitation Sedation Score; STAI: State-Trait Anxiety Inventory.

The intervention group will receive perioperative video goggles with an audio system designed to distract patients from the surgical process and enhance their experience. The video goggles will display various nature scenes accompanied by soft music. Patients can choose from a variety of preloaded content. The device meets the requirements of the Medical Device Directive 93/42/EWG and the Austrian Medical Devices Act. The anesthesiologists, who are primarily responsible for helping the patients use the goggles, received brief online training on how to operate the device. To minimize potential confounding factors, intraoperative management is performed by a small and experienced team of anesthesiologists. Figure 1 shows the timing and parameters that will be measured during surgery.

### Assessment of Distress

To assess patient stress, questionnaires and vital/laboratory parameters will be used. The day before surgery, after they provide informed consent, the patients will complete initial questionnaires. These include the Spielberger State-Trait Anxiety Inventory (STAI; Hogrefe Verlag), which measures both “trait anxiety” (a general predisposition to anxiety) and “state anxiety” (anxiety related to the current situation, ie, the upcoming surgery) [11]. This questionnaire is often used to measure anxiety and is seen as an indicator of distress. The score is used in many types of analysis, although the trait sections of the STAI are believed to measure negative affectivity rather than trait anxiety per se [12,13]. Additionally, patients will complete the Kurzfragebogen zur aktuellen Beanspruchung (KAB; German for “short questionnaire for acute stress”), which will be repeated perioperatively multiple times to assess changes in response patterns [14]. The focus will be on individual stress trajectories and differences between the control and intervention groups.

For a more objective measurement of stress, the cortisol level in the serum is measured by taking blood samples at multiple times (at baseline, arrival in the operation room, skin incision, clamping the carotid artery, skin suture, and 1 hour after skin suture). Baseline measurements are all taken under consistent conditions: the same time of day before the patient rises from bed. Resting heart rate, KAB score, and the Richmond Agitation-Sedation Scale (RASS) score will be evaluated simultaneously with blood sampling. Cortisol levels are a common tool to measure stress in acute situations [15-17]. The RASS, commonly used in anesthesiology and intensive care, will be used to estimate patients’ agitation and provide insight into their stress levels [18]. Heart rate and RASS scores serve as objective measures of patient stress, while the KAB reflects patients’ subjective burden across 6 categories. This score is frequently used in psychology to capture current stress levels and is well suited for perioperative use due to its ease of administration. The differences in serum cortisol levels between the groups are considered the primary end point. All other measurements are secondary end points.

### Assessment of Usefulness and Practicality

Postoperatively, the patients and the surgical team (the first surgeon and attending anesthesiologist) will complete a satisfaction survey using a 10-point numerical rating scale (NRS). Surgeons and anesthesiologists will rate the patients’ manageability, relaxation, and calmness during surgery. Patients will also rate the satisfaction and anxiety they experienced before and during surgery on a 10-point NRS scale. Patients in the video goggle group will be asked if they would choose to use the goggles again in a potential follow-up operation (as a dichotomic yes/no question). Motion sickness when using the goggles, a significant issue associated with immersion in virtual reality, will be assessed.

### Outcomes

#### Primary Feasibility Outcomes

Patients who meet the inclusion criteria and agree to participate are enrolled in the study. Patients who meet the criteria but decline participation are counted anonymously, with differentiation between those who refuse and those who are excluded for other reasons (eg, language barriers, profound hearing impairment, dementia, or logistical challenges). After the operation is completed and the patient leaves the wake-up room, all data are checked for completeness, and an attempt is made to fill in any missing data. If there any adverse events or remaining missing data, or if the patient withdraws consent to participate in the study, the collected data are excluded from analysis. Because of the very short time from patient inclusion to complete data collection for each patient and because there is no follow-up after the initial inpatient stay, we assume that this method will yield mostly complete records.

#### Clinical Outcomes

In addition to patient satisfaction, anxiety before and during surgery will be assessed. Comparison of patient satisfaction and anxiety measures between the intervention and control groups will be conducted. Additionally, patients in the intervention group will be asked at the end of the operation day whether they would choose to use the goggles again during potential follow-up surgery. For these patients, the average STAI score will be compared between those who would opt to use the goggles again and those who would not. Furthermore, comparisons of the recorded vital parameters and scores at different time points during surgery will be made between the control and intervention groups.

### Data Collection and Management

Missing data are not expected; however, if data are incomplete for a participant, the participant will be excluded from the analysis. Group allocation and reasons for exclusion will be reported transparently to minimize the risk of bias. Due to the pilot nature of the study, no imputation is planned.

Patient-specific data and collected parameters are recorded on paper case report forms and later manually entered into a Microsoft Excel database. The complete dataset will be transferred to an SPSS (version 28, IBM Corporation) database for analysis. Digital data are stored in a folder with restricted access on a protected server of the University Hospital Augsburg. Paper-based data are stored in a locked office at the study office. Final data access is only granted to primary investigators.

### Planned Analysis

The analysis will be conducted using SPSS. Due to the study’s exploratory nature, descriptive statistics will be applied. Appropriate measures of central tendency and variability will be used depending on the level of measurement. Graphical representations will be made using line graphs or box plots.

## Results

The study is registered on the ClinicalTrials.gov platform (NCT06704230). Patient recruitment commenced in September 2022 and should be completed within 3 to 4 years. At the time of this protocol’s final submission for publication, approximately 90% of the recruitment had been completed. After the including the 100th patient, the acquired data will be analyzed and the results will be published in a peer-reviewed journal within one year.

## Discussion

### Summary

To our knowledge, this is the first study to investigate the perioperative use of video goggles in patients undergoing carotid surgery under local anesthesia. Audiovisual distraction is commonly used in surgical procedures performed under local or regional anesthesia to reduce patient stress and discomfort [8,19,20]. This, in turn, may lead to reduced use of analgesics and sedatives, thereby minimizing the risk of complications and overdose [21]. In addition, such interventions can facilitate outpatient procedures, allowing patients to be discharged shortly after treatment without the need for extended postoperative monitoring because of sedation.

In vascular surgery—particularly carotid endarterectomy performed under local anesthesia—continuous monitoring of the patient is essential to detect signs of cerebral hypoperfusion when the carotid artery is clamped. Local or regional anesthesia thus provides an advantage [22]. However, for the patient, undergoing surgery in the neck region can be physically and psychologically challenging, as they must remain in a fixed position on the operating table for approximately 2 hours while being covered by a sterile drape, which is placed closely over the patient’s face and cannot be repositioned without risking a breach of sterility. Patient stress during procedures under local anesthesia can also pose a challenge for the clinical team and affect surgical outcomes. The current literature does not indicate a significant overall benefit of one anesthetic technique over the other in carotid endarterectomy; thus, the decision should be individualized [4]. This study aims to assess the feasibility of using video goggles during such procedures and to evaluate their potential in reducing patient-reported stress levels. Furthermore, there is currently no tool to predict which patients may benefit from the use of video goggles and which may not.

### Strengths, Limitations, and Dissemination

To evaluate patient stress, we use a multimodal approach that includes objective and subjective parameters. Serum cortisol levels serve as the primary objective marker, complemented by heart rate monitoring and the RASS. Additionally, the KAB questionnaire was used to assess patient-perceived stress. All parameters were recorded at multiple time points before, during, and after the operation, enabling a comparative analysis comparing the intervention and control groups.

Drug administration was meticulously documented, as medications could potentially confound the measured parameters. Although the sample size is limited, which may preclude statistically significant findings, and this is a single-center study, which means that just one device in one specific procedure is evaluated, the study primarily aims to assess the feasibility and acceptance of video goggles by patients, anesthesiologists, and surgeons, as well as to identify robust parameters for future research. By publishing our findings in a peer-reviewed journal, we hope to provide a basis for further research in this field and to promote enhanced approaches to perioperative stress reduction in patients undergoing carotid surgery under local anesthesia. In future work, a potential third group using music as a distraction tool could be explored.

### Conclusions

This pilot study explores the implementation of video goggles during carotid surgery under conscious sedation and aims to identify suitable outcome parameters for evaluating this interventin’s potential benefits in reducing perioperative stress.

## Abbreviations

KAB: Kurzfragebogen zur aktuellen Beanspruchung; German for “short questionnaire for acute stress”
NRS: numeric rating scale
RASS: Richmond Agitation-Sedation Scale
STAI: Spielberger State-Trait Anxiety Inventory
